# For how many days and what types of group activities should older Japanese adults be involved in to maintain health? A 4-year longitudinal study

**DOI:** 10.1371/journal.pone.0183829

**Published:** 2017-09-14

**Authors:** Kumiko Nonaka, Hiroyuki Suzuki, Hiroshi Murayama, Masami Hasebe, Takashi Koike, Erika Kobayashi, Yoshinori Fujiwara

**Affiliations:** 1 Research Team for Social Participation and Community Health, Tokyo Metropolitan Institute of Gerontology, Itabashi-ku, Tokyo, Japan; 2 Institute of Gerontology, The University of Tokyo, Bunkyo-ku, Tokyo, Japan; 3 Faculty of Human Welfare, Seigakuin University, Ageo-shi, Saitama, Japan; 4 Faculty of International Studies of Culture, Kyushu Sangyo University, Higashi-ku, Fukuoka, Japan; University of West London, UNITED KINGDOM

## Abstract

**Objective:**

Studies have suggested that frequent participation in social groups contributes to the well-being of older people. The primary aim of this study was to identify the number of days older adults should participate in the activities of social groups to maintain their health for 4 years. This study also aimed to examine whether the effective frequency differs by the type of social group activity.

**Method:**

We examined a prospective cohort of 1,320 community-dwelling older adults over 65 years of age, who responded to both a baseline and a follow-up mail survey, in a suburban city of Tokyo, Japan. The dependent variable was the change in functional competence during 4 years. Logistic regression analyses were conducted to examine the effects of participation in the activities of the 5 most common social groups among older Japanese on maintaining functional competence.

**Results:**

Nine hundred and ninety-four participants (76.5%) maintained their functional competence for 4 years. The results of the logistic regression analyses showed that participating in alumni groups less than once a month and being an inactive member were associated with higher odds of maintaining functional competence, after controlling for socioeconomic, demographic and baseline health status. Additionally, the odds of maintaining functional competence for 4 years increased upon participating in volunteer groups once a month or more. These results were also confirmed using logistic regression analysis, even after adjustment for the effects of participation in other social groups.

**Discussion:**

The results indicated the effectiveness of volunteer activities that fulfill a social role in maintaining health. Therefore, older adults should be encouraged to participate in activities of volunteer groups at least once a month. Additionally, older adults can obtain positive health outcomes through less frequent participation in alumni groups, compared with the activities of volunteer groups.

## Introduction

Older adults have been encouraged to engage in social and productive activities, particularly in activities of social groups, in Japan [1]. In 2013, 61% of older Japanese adults, over the age of 60 years, participated in some kinds of social group activities, and this participation rate was 6.2 points higher than in 2003 [2]. It has been suggested that the involvement in community or social groups contributes to the physical and psychological well-being of older adults, by providing them with emotional and material support [3–7], as well as giving them a sense of purpose [3, 4]. For example, a previously conducted study showed that the perceived health status and activities of daily living (ADL) were improved by participation in local social, political, religious, sports, and school-related groups or organizations [8]. Participation in the activities of volunteer groups was also reported to be beneficial to the cognitive, physical, and psychological health of older adults [9–11].

Several studies have examined whether health outcomes differ by the types of activities. Oman *et al*. [12] found that the reduction in mortality associated with volunteering was larger than the reduction associated with physical mobility, exercising, and attendance at religious services [12]. In addition, Minagawa *et al*. [13] examined whether there were differences in health consequences by the types of activities, such as involvement in neighborhood associations, senior clubs, educational/sports/hobby groups, and volunteer groups. Their findings showed that activities that were geared more towards self-development, such as postretirement employment and lifelong learning, had strong associations with lower levels of mortality [13].

In addition to the types of activities, earlier studies have pointed out the importance of engagement frequency. Frequent physical and social activities were reported to have protective effects on dementia [14–19]. Similarly, increased hours of engagement in volunteer activities had protective effects on dementia incidence [20], improved life satisfaction [21, 22], reduced depressive symptoms [22, 23], contributed to higher levels of self-rated health and reduced functional dependency [23], and reduced mortality [16]. At the same time, several previously conducted studies have pointed out that the positive effects of volunteer activities are limited to certain amounts of time [21, 23]. For example, Morrow-Howell [22] reported that older adults who engage in longer hours of volunteering had a better sense of well-being; but, the positive impact of volunteering on health was limited to a maximum of 100 hours a year.

Although previous studies have shown the benefits of frequent participation in the activities of social and volunteer groups, as well as the time limits of engaging in volunteer activities for beneficial outcomes, there is little knowledge on how often or how many days older adults should engage in the activities of social groups to maintain their health. In addition, only a limited number of studies has examined whether effective engagement frequency differs by the types of activities. Therefore, the first aim of this study was to demonstrate how many days older adults should participate in the activities of social groups in order to maintain their health. The second aim of this study was to examine whether effective frequency differs by the types of different social groups’ activities. We analyzed the participation in the activities of 5 types of social groups, which were most common among older Japanese adults. This study focused on the number of days, as this may provide a clear standard for older adults to understand how often they should engage in social activities, in a month or year, to maintain their health. Thus, the present study aimed at proposing how many days older adults should be involved in what types of group activities to maintain their health.

## Materials and methods

### Participants

A 4-year longitudinal study of older adults, aged 65 years and above, was conducted in 2008 and 2012 in City A. City A is a typical commuter city, located in the suburbs of Tokyo, Japan. It had a total population of 74,879 in 2008, with 7,100 persons/km^2^. Adults who were 65 years old and older accounted for 13.4% (10,003) of the total population. A baseline mailed survey (T1) was conducted between July and December 2008. First, 2,600 adults over the age of 65 years were randomly selected from among residents of the city, on July 1, 2008. We excluded 72 participants because of moving or death. Thus, the final sample of the T1 consisted of 2,528 older adults. The exclusion criteria of the sample were older adults living in institutional care facilities and being registered as nursing Care-Level 2 or more severe, which was equivalent to a condition requiring assistance in basic activities of daily living (ADL), such as using the toilet or eating. We collected replies from 1,773 participants (70.1%) of the T1.

A follow-up mailed survey (T2) was conducted on July 1, 2012, including all 11,172 residents of the city, who were 65 years old and above. The exclusion criteria of the sample were the same as in T1, and we collected replies from 8,297 (74.2%) residents. From among the 1,773 participants of T1, 449 did not participate in T2 because of moving (n = 63), institutionalization (n = 44), death (n = 77), and refusal or an unknown reason (n = 265). Four participants were excluded from the analyses because of incomplete information on the questionnaire, relating to functional competence. Therefore, we analyzed the data of the 1,320 residents who replied to both T1 and T2.

Only those who agreed to participate in the study returned the questionnaire; this was regarded as consent to participate in the study. The study protocol was approved by the Institutional Review Board of the Tokyo Metropolitan Institute of Gerontology (TMIG). The sampling method of the study has been provided elsewhere [24].

### Measurements

The dependent variable was the change in functional competence, between T1 and T2. Functional competence was measured by the Tokyo Metropolitan Institute of Gerontology Index of Competence (TMIG-IC) [25]. The TMIG-IC is a multidimensional 13-item index which was developed to grade the functional competence of older people with intact ADL, according to their abilities to perform activities more complex than ADL. The response to each item was scored as 0 for “unable to do” and 1 for “able to do”. The total TMIG-IC score (0–13) was composed of the three subscales of instrumental ADL (IADL), intellectual activity, and social role. Higher scores indicated better performance. We created a dichotomous variable of change in functional competence (CFC) as the dependent variable, which consisted of two categories: “declined” and “maintained”. The “declined” category consisted of participants whose TMIG-IC scores in T2 decreased by 2 or more points from T1, because the test-retest method demonstrated that variations of 2 points and over for total scores meant a significant change in functional capacity among older people [26]. The “maintained” category included participants whose TMIG-IC scores in T2 were unchanged, improved, or decreased by 1 point from T1.

The social groups of this study included: (1) alumni groups, (2) neighborhood associations, (3) senior clubs, (4) hobby/sports/culture groups, and (5) volunteer groups. Alumni groups, in this study, refer to both school and work-related alumni groups. Respondents were asked if they had participated in each type of social group’s activity in the past year and then, they were asked about their frequency of participation in each social group. The response category of frequency consisted of: (1) do not belong (non-member), (2) have membership but did not participate in the past year (inactive member), (3) participated less than once a month, and (4) participated once a month or more.

The confounding variables were age, gender, self-reported economic status, years of education, self-rated health, and baseline functional competence. The self-reported economic statuses of the participants comprised: “poor”, “neither poor nor wealthy”, and “wealthy”. The years of education were categorized as “less than 13 years” and “13 years and over”, which was equivalent to high school education and above. The baseline functional competence was assessed by the total score of the TMIG-IC. The self-rated health status was a dichotomous variable of “good” and “fair/poor”.

### Data analysis

First, we analyzed the differences in the baseline characteristics between the “maintained” and “declined” categories, using the chi-square test or Fisher’s exact test (when the expected number was <5) for categorical variables, and the t-test for continuous variables in assessing the statistical significance of the differences, where p<0.05.

Second, 5 models of logistic regression analyses were conducted to evaluate the independent effect of participation in each group’s activity on functional competence; non-members were the reference group. In each analysis, the confounding variables were entered into the model after we took the degree of multiple colinearity into consideration.

Third, logistic regression analysis was conducted, entering all the variables of participation in the social groups, which obtained a higher level of significance lower than 0.05, at the second stage of the analyses. This analysis was conducted to confirm the effectiveness of participation in each social group’s activity, after controlling the effects of participation in other groups’ activities. Statistical analyses were performed using SPSS (version 23; SPSS Japan Inc., Tokyo, Japan) [S1 Dataset]. The level of significance was set at <0.05.

## Results

Table 1 and Table 2 show the baseline characteristics of the “declined” and “maintained” categories [Tables 1 and 2]. While 994 study participants (76.5%) maintained functional competence for 4 years, a decline in functional competence was observed in 306 (23.5%) participants. Participants who were categorized as “maintained” were likelier to be younger (71.5±4.9) and female (53.9%), have a higher educational level and a higher score in the TMIG-IC, and have participated in each activity more frequently than the “declined” group. In terms of the relationship between the participation in each social group and its frequency, those in the “maintained” category participated in the activities of volunteer and hobby/sports/culture groups (6.7% and 32.5%, respectively) once a month or more than the “declined” category (1.4% and 20.3%, respectively). Similarly, a larger number of respondents who were categorized as “maintained” participated in activities of neighborhood associations and alumni groups (19.4% and 26.5%, respectively) less than once a month, compared to the “declined” category (19.4% and 36.7%, respectively).

**Table 1:** Baseline characteristics of the study participants and their association with change in functional competence (2008–2012).

	Characteristic	Maintained^a^	Declined^b^	*P*-value^c^
		(n = 994)	(n = 306)	
Age, years, mean ± SD		71.5 ± 4.9	74.6 ± 6.1	< .001
Gender, female, %		53.9	45.8	.013
Self-reported economic status				
	poor, %	30.2	37.0	.540
	neither poor nor wealthy, %	43.7	42.2	
	wealthy, %	26.1	20.8	
Years of education				
	less than 13 years, %	70.7	77.1	.052
	13 years and over, %	29.3	22.9	
Self-rated health,				
	poor or fair, %	14.9	26.7	.000
	good, %	85.1	73.3	
Total scores of TMIG-IC, mean ± SD		11.6 ± 2.2	11.4 ± 2.0	
Alumni group				
	non-member, %	43.2	57.5	.000
	inactive member, %	17.3	14.3	
	participated less than once a month, %	36.7	26.5	
	participated once a month or more, %	2.7	1.7	
Neighborhood association				
	non-member, %	32.6	36.1	.004
	inactive member, %	29.1	36.8	
	participated less than once a month, %	28.3	19.4	
	participated once a month or more, %	10.0	7.6	
Senior club				
	non-member, %	84.8	80.1	.030
	inactive member, %	4.2	6.8	
	participated less than once a month, %	5.6	9.1	
	participated once a month or more, %	5.4	4.1	

*p < .05.

**p < .01.

^a^ 4-year change in functional competence assessed according to the TMIG-IC classification, based on the difference between 2008 and 2012 TMIG-IC scores.

^b^ Decrement of 2 points in 4 years.

^c^ P-values from chi-square tests or Fisher’s exact test (when the expected number was <5) for categorical variables and t-test for continuous variables.

**Table 2:** Baseline characteristics of the study participants and their association with change in functional competence (2008–2012). Continued.

	Characteristic	Maintained^a^	Declined^b^	*P*-value^c^
		(n = 994)	(n = 306)	
Hobby/sports/culture group				
	non-member, %	53.6	65.5	.001
	inactive member, %	2.1	2.8	
	participated less than once a month, %	11.7	11.4	
	participated once a month or more, %	32.5	20.3	
Volunteer group				
	non-member, %	84.4	91.0	.005
	inactive member, %	1.5	1.4	
	participated less than once a month, %	7.4	6.3	
	participated once a month or more, %	6.7	1.4	

*p < .05.

**p < .01.

^a^ 4-year change in functional competence assessed according to the TMIG-IC classification, based on the difference between 2008 and 2012 TMIG-IC scores.

^b^ Decrement of 2 points in 4 years.

^c^ P-values from chi-square tests or Fisher’s exact test (when the expected number was <5) for categorical variables and t-test for continuous variables.

Table 3 and Table 4 show that participation in alumni groups less than once a month and being an inactive member were associated with higher odds of maintaining functional competence for 4 years (OR = 1.82, 95% CI = 1.20–2.77; OR = 1.65, 95% CI = 1.02–2.67, respectively) [Tables 3–4]. In addition, the odds of maintaining functional competence for 4 years increased by participation in the activities of volunteer groups once a month or more (OR = 3.27, 95% CI = 1.14–9.38) [Table 4]. On the other hand, participation in senior clubs less than once a month was negatively associated with maintaining functional competence (OR = .05, 95% CI = 0.28–0.96) [Table 3]. The protective effects of participation in hobby/sports/culture groups and neighborhood associations disappeared when the confounding variables were entered into the analyses [Table 4].

**Table 3:** Adjusted odds ratios for predicting improvement or maintenance of functional competence during the 4-year follow-up period, by 5 models (2008–2012).

Model			OR	95%C.I.
1.	Age, an increment by 1 year		0.91	0.89–0.94**
	Female (vs. male)		2.00	1.40–2.86*
	Self-reported economic status (vs. poor)			
		neither poor nor wealthy	1.18	0.80–1.75
		Wealthy	1.13	0.72–1.77
	Education, 13 years and over (vs. less than 13 years)		1.29	0.86–1.96
	Self-rated health, good (vs. poor or fair)		1.56	1.01–2.39*
	Total scores of TMIG-IC, increment by 1 points		0.96	0.88–1.05
	Alumni (vs. non-member)			
		inactive member	1.65	1.02–2.67*
		participated less than once a month	1.82	1.20–2.77**
		participated once a month or more	2.30	0.64–8.29
2.	Age, an increment by 1 year		0.92	0.89–0.95**
	Female (vs. male)		1.83	1.29–2.60**
	Self-reported economic status (vs. poor)			
		neither poor nor wealthy	1.14	0.76–1.69
		Wealthy	1.14	0.72–1.81
	Education, 13 years and over (vs. less than 13 years)		1.44	0.97–2.15
	Self-rated health, good (vs. poor or fair)		1.47	0.95–1.09
	Total scores of TMIG-IC, increment by 1 points		1.00	0.92–1.09
	Neighborhood association (vs. non-member)			
		inactive member	0.76	0.50–1.15
		participated less than once a month	1.15	0.71–1.85
		participated once a month or more	0.96	0.51–1.82
3.	Age, an increment by 1 year		0.91	0.89–0.94**
	Female (vs. male)		1.83	1.30–2.59**
	Self-reported economic status (vs. poor)			
		neither poor nor wealthy	1.15	0.78–1.69
		Wealthy	1.25	0.80–1.96
	Education, 13 years and over (vs. less than 13 years)		1.48	1.00–2.19
	Self-rated health, good (vs. poor or fair)		1.47	0.96–2.26
	Total scores of TMIG-IC, increment by 1 points		1.00	0.92–1.08
	Senior club (vs. non-member)			
		inactive member	1.12	0.48–2.60
		participated less than once a month	0.52	0.28–0.96*
		participated once a month or more	1.44	0.64–3.25

*p < .05.

**p < .01.

**Table 4:** Adjusted odds ratios for predicting improvement or maintenance of functional competence during the 4-year follow-up period by 5 models (2008–2012). Continued.

Model			OR	95%C.I.
4.	Age, an increment by 1 year		0.91	0.89–0.94**
	Female (vs. male)		1.70	1.19–2.41**
	Self-reported economic status (vs. poor)			
		neither poor nor wealthy	1.14	0.77–1.68
		Wealthy	1.17	0.74–1.83
	Education, 13 years and over (vs. less than 13 years)		1.45	0.97–2.17
	Self-rated health, good (vs. poor or fair)		1.41	0.92–2.17
	Total scores of TMIG-IC, increment by 1 points		0.99	0.91–1.08
	Hobby/sports/culture group (vs. non-member)			
		inactive member	0.74	0.25–2.24
		participated less than once a month	1.05	0.62–1.79
		participated once a month or more	1.41	0.93–2.14
5.	Age, an increment by 1 year		0.91	0.89–0.94**
	Female (vs. male)		1.85	1.30–2.62**
	Self-reported economic status (vs. poor)			
		neither poor nor wealthy	1.15	0.77–1.70
		Wealthy	1.17	0.74–1.84
	Education, 13 years and over (vs. less than 13 years)		1.44	0.96–2.14
	Self-rated health, good (vs. poor or fair)		1.51	0.99–2.32
	Total scores of TMIG-IC, increment by 1 points		1.00	0.91–1.08
	Volunteer group (vs. non-member)			
		inactive member	1.51	0.32–7.07
		participated less than once a month	0.93	0.49–1.76
		participated once a month or more	3.27	1.14–9.38*

*p < .05.

**p < .01.

Logistic regression analysis was conducted by entering the variables of participation in the activities of volunteer and alumni groups, with non-members constituting the reference group. Table 5 shows that the odds of maintaining functional competence for 4 years increased among those who participated in the activities of volunteer groups once a month or more (OR = 2.98, 95% CI = 1.03–8.66) [Table 5]. Similarly, participation in alumni groups less than once a month and being an inactive member increased the possibility of maintaining functional competence compared to non-members (OR = 1.98, 95% CI = 1.28–3.06; OR = 1.67, 95% CI = 1.03–2.72, respectively) [Table 5].

**Table 5:** Adjusted odds ratios of the types of social activity for predicting the improvement or maintenance of functional competence for 4 years, by entering the social activities obtained p<0.05.

Model		OR	95%C.I.
Age, an increment by 1 year		0.91	0.89–0.94**
Female (vs. male)		2.00	1.40–2.86**
Self-reported economic status (vs. poor)			
	neither poor nor wealthy	1.18	0.80–1.75
	Wealthy	1.13	0.72–1.77
Education, 13 years and over (vs. less than 13 years)		1.29	0.86–1.96
Self-rated health, good (vs. poor or fair)		1.56	1.01–2.39*
Total scores of TMIG-IC, increment by 1 points		0.96	0.88–1.05
Alumni group (vs. non-member)			
	inactive member	1.67	1.03–2.72*
	participated less than once a month	1.98	1.28–3.06**
	participated once a month or more	2.12	0.58–7.75
Volunteer group (vs. non-member)			
	inactive member	1.39	0.30–6.51
	participated less than once a month	0.75	0.39–1.44
	participated once a month or more	2.98	1.03–8.66*

*p < .05.

**p < .01.

## Discussion

The present study explored how many days older adults should participate in the activities of social groups in order to maintain their functional competence, and whether effective participation frequency differed by the types of activities. Bivariate analyses of the baseline characteristics indicated that those who maintained their functional competence tended to participate in the activities of social groups more frequently than those who showed a decline in functional competence. However, the protective effects of participating in hobby/sports/culture groups and neighborhood associations disappeared when confounding variables were entered into the logistic regression analyses. On the other hand, the results of the logistic regression analyses showed that participation in the activities of volunteer groups was effective in maintaining functional competence, after controlling for confounding variables. Similarly, the odds of maintaining functional competence for 4 years increased upon participating in the activities of alumni groups less than once a month or being an inactive member.

In terms of hobby/sports/culture groups, the results were inconsistent with those of previous studies which demonstrated the protective effects of leisure, hobby, sports, culture, and continued learning activities on the well-being of older adults [15, 20, 27, 28]. These results indicate that factors other than engaging in these group activities were more influential on the well-being of the participants in this study. Indeed, several studies reported that older adults with a higher educational attainment were more likely to participate in leisure and self-development activities, such as continued learning [15, 29]. Health status and age were also reported to influence participation, as older age and physical health problems reduced the frequency of participation in any kind of activity, including leisure activities [15, 29].

The results of this study demonstrated that participating in the activities of volunteer groups once a month or more was particularly effective in maintaining functional competence for older adults, compared with other types of social group activities. The result was also confirmed by logistic regression analysis, controlling for socioeconomic, demographic and baseline health variables, as well as participation in alumni groups. This result was consistent with that of previous studies, in which the reduction in mortality associated with volunteering was larger than the reduction associated with physical mobility, exercising, and attendance at religious services [12]. This study added data about the number of days that older adults should engage in the activities of volunteer groups, to maintain functional competence.

Although the causal mechanism of how volunteering affects health remains unclear, our results can be interpreted as the effectiveness of the activities that fulfill a social role function, for the well-being of older adults. Lawton [30] conceptualized 7 stages of competence, which were arranged from the most basic function to higher functions, in an ascending order of complexity. Life maintenance is the most basic competence, and the other competences advance in the order of functional health, perception and cognition, physical self-maintenance, IADL, effectance that is equivalent to intellectual ability, and social role. Fujiwara *et al*. [26] supported this conceptualization, and demonstrated that older adults with no initial disability at the baseline were the most likely to lose social role function with advancing age, followed by intellectual ability and IADL. Volunteer activities involve motivations, such as making a contribution to others or to society [9, 31] and acts toward “clients”. These characteristics of volunteer activity may provide individuals with a social role, which in turn contributes to the physical well-being of older adults. Therefore, older adults should be encouraged to participate in activities of volunteer groups at least once a month.

Additionally, the study showed that older adults were more likely to maintain functional competence through participation in the activities of alumni groups less than once a month, or as an inactive member. The insignificance of participation in alumni groups once a month or more may be attributed to the smaller number of respondents who participated in alumni groups once a month or more. Indeed, alumni groups in Japan usually offer infrequent activities, such as having monthly or annual gatherings. Although there were a few studies that examined how alumni groups affect individuals’ health, it can be assumed that the type of network influences the individuals’ health. Members of alumni groups may consist of relatively similar demographic and socioeconomic characteristics. Thus, alumni groups may provide individuals with bonding social capital, which refers to “horizontal ties between members of a network who see themselves as similar” [32]. A study reported that bonding social capital contributed to better self-rated health, over and above the beneficial effects of social networks and support [32]. Therefore, older adults can obtain positive health outcomes through less frequent participation in alumni groups, compared with the activities of volunteer groups.

This study has several limitations. First, it could not include other factors, such as the motives of participation and the roles the individuals played within a group. Previous studies pointed out that the protective effect of frequent participation in social activities was limited to individuals with other-oriented motivation [10] and who performed a key role in an organization [33]. Second, this study failed to examine the effects of participation in multiple types of activities and groups. Previous studies indicated that engaging in volunteer activities in multiple organizations, increased life satisfaction and perceived health, compared to engaging in a single organization [20]. Conversely, another study suggested that the number of sponsoring organizations was not related to well-being outcomes. Finally, this study could not elucidate the differences in the health consequences among older adults who engaged in activities more than once a month, because relatively small numbers of participants engaged in the activities of volunteer groups.

In conclusion, participating in the activities of volunteer groups once a month or more was particularly effective in maintaining functional competence for older adults, compared to other types of social group activities, by stimulating a social role. Additionally, older adults were more likely to maintain their functional competence through participation in the activities of alumni groups less than once a month or as an inactive member.

## Supporting information

S1 Dataset SPSS. SPSS dataset.
(ZIP)
